# Movable Kidney

**Published:** 1904-06

**Authors:** 


### Movable Kidney.

Rufus B. Hall, Cincinnati, (*Am. Jour. of Obstetrics,* Jan., 1904,) says the present practice, in this condition, is not to interfere until compelled to do so by torsion of the pedicle, or some serious pathological condition. While it may be conceded that only a small percentage of patients suffering from movable kidney have obstruction in the ureter, necessitating an operation for relief, Hall believes that quite a large proportion develop hydro- or pyonephrosis, and in a few, complete obstruction. Many of these cases suffer more or less discomfort in the region of the displaced kidney and annoyance. The pain is often constant so long as the patient is in the upright position. Fixation gives these patients relief. The author is of the opinion that these cases should be operated upon before hydronephrosis may develop, as the gradually increasing curvature of the ureter with its obstruction has a marked effect upon the excretory function of the kidney and may eventually produce atrophy and cystic degeneration. The urine being unable to escape, it distends the pelvis and calices, causing destruction in the tubules, and the Malpighian tufts, and terminates in a cyst, the walls of which are composed of the pelvis and the capsule of the kidney. During the acute attacks, the examination of urine will reveal more or less pus present; but this will almost entirely disappear three or four days after the attack has subsided. This examination of the urine between attacks may cause a faulty diagnosis. The operation of relief of this condition, is almost devoid of danger; and, if it is performed before serious pathological changes have taken place in the organ, relief is immediate and permanent.—*Medical News.*

